# Does aquatic physical therapy affect the rehabilitation of breast cancer in women? A systematic review and meta-analysis of randomized controlled trials

**DOI:** 10.1371/journal.pone.0272337

**Published:** 2022-08-03

**Authors:** Juzi Wang, Xiaoyu Chen, Lili Wang, Caiyun Zhang, Ji Ma, Qian Zhao

**Affiliations:** 1 Department of Nursing, Shanxi Provincial People’s Hospital, Taiyuan, Shanxi, China; 2 School of Nursing, Shanxi University of Traditional Chinese Medicine, Jinzhong, Shanxi, China; 3 The Orthopaedic Spinal Ward, Shanxi Provincial People’s Hospital, Taiyuan, Shanxi, China; Universidade Federal da Bahia, BRAZIL

## Abstract

To determine and evaluate the benefits of aquatic physical therapy as a rehabilitation strategy for women with breast cancer on health outcomes. Electronic databases including PubMed, Web of Science, Embase, Cochrane Library and China National Knowledge Infrastructure (CNKI), Weipu (VIP) and Wanfang database were systematically searched until June 2021. Randomized controlled trials were included if they evaluated the effects of aquatic physical therapy in breast cancer patients. The quality of the trials included was assessed by the two independent researchers according to the Cochrane Collaboration Handbook recommendations. Outcome measures were fatigue, waist circumference and quality of life (QoL). The study was registered under PROSPERO (CRD42021157323). Totally, five studies comprising 356 participants were included in the study. Meta-analyses showed that aquatic physical therapy interventions significantly reduced the fatigue score (MD = -2.14, 95%CI: -2.82, -1.45, *p*<0.01) compared with usual care; In addition, we also observed that, compared with land-based exercise, aquatic physical therapy greatly improved the QoL (MD = 2.85, 95%CI: 0.62, 5.09, *p* = 0.01). However, aquatic physical therapy cannot improve physical index (waist circumference) compared to usual care (MD = -3.49, 95%CI: -11.56,4.58, *p* = 0.4). Consequently, aquatic physical therapy had a positive effect on the fatigue and QoL. The results of this meta-analysis can provide a reliable evidence for evaluating the interventional effectiveness of aquatic physical therapy.

## Introduction

Breast cancer is the most prevalent cancer among women worldwide. Each year more than 1.7 million women are diagnosed and more than 500,000 die from breast cancer, making it the leading cause of cancer death among women globally [1]. However, a recent report shows that the 5-year survival rate of breast cancer has increased significantly in the past three decades, exceeding 90%, and will continue to increase [2].

Advances in breast cancer detection and treatment have resulted in improved survival rates and thereby a growing number of cancer survivors, and the QoL is getting more and more attention [3]. High survival rates are often accompanied by many side effects caused by cancer and related treatments [4], such as lymphedema, fatigue, and decreased QoL, muscle strength and endurance [5, 6]. These effects may be short-term, or they may appear only a few years after treatment and persist for a long time. Therefore, breast cancer survivors need a measure to cope with their long-term health needs.

As early as 2010, the American College of Sports Medicine has formulated exercise guidelines for cancer survivors, suggesting that exercise training is safe during and after cancer treatment and can improve the physical function, quality of life and cancer-related fatigue in cancer groups including breast cancer [7].

Aquatic physical therapy—also known as hydrotherapy or aquatic exercise [8]—has been widely used for the purpose of rehabilitation and treatment of many diseases [9], such as rheumatic disease, fibromyalgia, stroke, Parkinson disease, and so on [10–12]. Aquatic physical therapy is defined as exercise in lukewarm water, assistance and resistance of warm water to relieve pain, muscle relaxation and making more effective exercise, which is a safe and effective medium treatment way for achieving exercise-related goals [13, 14]. In general, this therapy involves a variety of exercise modalities including aerobic, stretching, resistance, flexibility and stability training [15]. By using the unique properties of water (buoyancy, resistance, flow, and turbulence), aquatic physical therapy allows people to perform exercises that they cannot do on land, and can create a progressive exercise program for breast cancer survivors from auxiliary exercises to resistance exercises [16].

In addition, studies have shown that aquatic physical therapy not only reduces the lymphedema risk in those with breast cancer [17], but also relieves persistent skin dryness caused by cancer treatment and significantly decreases the incidence of skin induration, thereby potentially decreasing chances of cancer recurrence [18]. Meanwhile, it provides a safe, effective and non-invasive method for the treatment of breast cancer survivors, and avoids invasive methods and oral medications that cannot be tolerated over a long period due to their adverse systemic effects [19]. In light of this, aquatic physical therapy would be an ideal form of physical rehabilitation for patients with breast cancer.

Despite these recent findings, for breast cancer survivors, 56–95% of patients experience cancer-related fatigue (CRF) after treatment [20], and nearly 20% of these patients may persist for several years [21], which reduces the patient’s QoL to a certain extent [22]. At the same time, chemotherapy also affects metabolic changes in the muscle, resulting in a significant reduction in skeletal muscle mass or an increase in body fat, which in turn has a lasting effect on the patient’s physical index, and these changes translate into a threat of cancer recurrence [17]. However, to our knowledge, there was no relevant systematic reviews to evaluate the effects of aquatic physical therapy on the above changes. Therefore, it is necessary to systematically evaluate the effects of aquatic physical therapy on fatigue, QoL, and physical index in breast cancer patients. For this purpose, we performed a systematic review and meta-analysis of randomized controlled trials (RCTs) to better understand the current evidence on the effects of aquatic physical therapy as a rehabilitation strategy in breast cancer to provide further evidence for the management of breast cancer survivors.

## Materials and methods

This is a meta-analysis of randomized trials involving the effects of aquatic physical therapy on fatigue, QoL and physical index in women with breast cancer. The systematic review and meta-analysis were reported in accordance with the recommendations of the Preferred Reporting Items for Systematic Review and Meta-Analyses: The PRISMA Statement and Cochrane Handbook for Systematic Reviews of Interventions [23, 24]. The selected search strategy and methods of analysis were registered at the PROSPERO database (ref: CRD42021157323).

### Search strategy

The search strategy was formulated by the PICO structure in accordance with the PRISMA statement [23]. Participants (P) included adults (≥ 18 years old) women with breast cancer who were required to finish co-adjuvant treatment and were not considered to have any contraindications to limited activity. Interventions (I) included aquatic physical therapy and its various forms of exercise including physical activity, strength training, aerobic exercise, *etc*. Comparisons (C) included all forms of intervention except aquatic exercise. Outcomes (O) included improvements in fatigue, QoL, and physical index. Study design included RCTs.

We searched the following databases including Medline/PubMed, Web of Science, Embase, Cochrane Library and Chinese databases of the CNKI Scholar, VIP and Wanfang. The relevant studies were searched from the inception of each database to June 2021. The search terms and strategy used were as follows: (hydrotherapy OR aquatic exercise OR water-based exercise) AND (breast neoplasms OR breast cancer) AND (randomized controlled trial OR RCT). Additionally, to search all relevant studies, the reference lists were also manually reviewed. The detailed search strategy is available in the S1 Appendix.

### Inclusion and exclusion criteria

The study inclusion criteria were: (1) participates were women with a clinical diagnosis of breast cancer; (2) participants aged ≥18 years; (3) participants have finished co-adjuvant treatment; (4) during the intervention period, participants did not have any contraindications limiting their activities; (5) at least one group of intervention methods was aquatic physical therapy involving any form of exercise in lukewarm water; (6) the study was reported at least one of the outcomes: fatigue, QoL, and physical index; (7) the type of study design was the RCT. Studies were excluded if (1) the type of article was conference abstracts, case reports, comments, letters to editor, review articles, and family-based studies; (2) the full text of the study was not available; (3) studies without available data; (4) the type of study design was not the RCT.

### Data extraction and quality assessment

Two independent researchers have screened all abstracts identified in the initial search, excluded studies that violated the inclusion criteria, and have removed all the duplicated references. If it was unclear whether the study met the selection criteria, advice could be sought from a third researcher and a consensus of opinion was made.

Information on first author and publication year, country, cancer stage, sample size, age and interventions in all study arms, and outcomes measures were extracted from the original reports. The quality of the trials included was assessed by the two independent researchers according to the Cochrane Collaboration Handbook recommendations and items such as: randomization, allocation concealment, blinding, incomplete outcome data and selective reporting [24]. It means low risk if the thesis clearly described, high risk if not described and unclear if described indeterminately in the text. Researchers achieved consensus by discussion, and if researchers didn’t achieve, a third reviewer was consulted.

### Outcome measures

The outcomes of fatigue and waist circumference between aquatic physical therapy and usual care were analyzed. The QoL was analyzed to show whether aquatic physical therapy could improve score compared land-based exercise after 1 year of intervention.

### Statistical analysis and risk of bias assessment

The data were analyzed by RevMan software (Version 5.4.1). A meta-analysis intended to be carried out on RCTs, if the same outcomes had been assessed in at least two studies in a similar way, and at least one group of received aquatic physical therapy. The mean difference (MD) and 95% confidence interval (CI) were calculated for continuous data to assess the change. The heterogeneity among studies was assessed by *I*^*2*^. If *I*^*2*^*<*50%, it could be considered that there was homogeneity among the trials, and the fixed-effects model was used; otherwise, a random-effects model was used (*I*^*2*^*≥*50%). A z test was adopted to test the combined effect and statistical significance was set at *p<*0.05 [25].

## Results

### Study selection and characteristics

**Fig 1:**
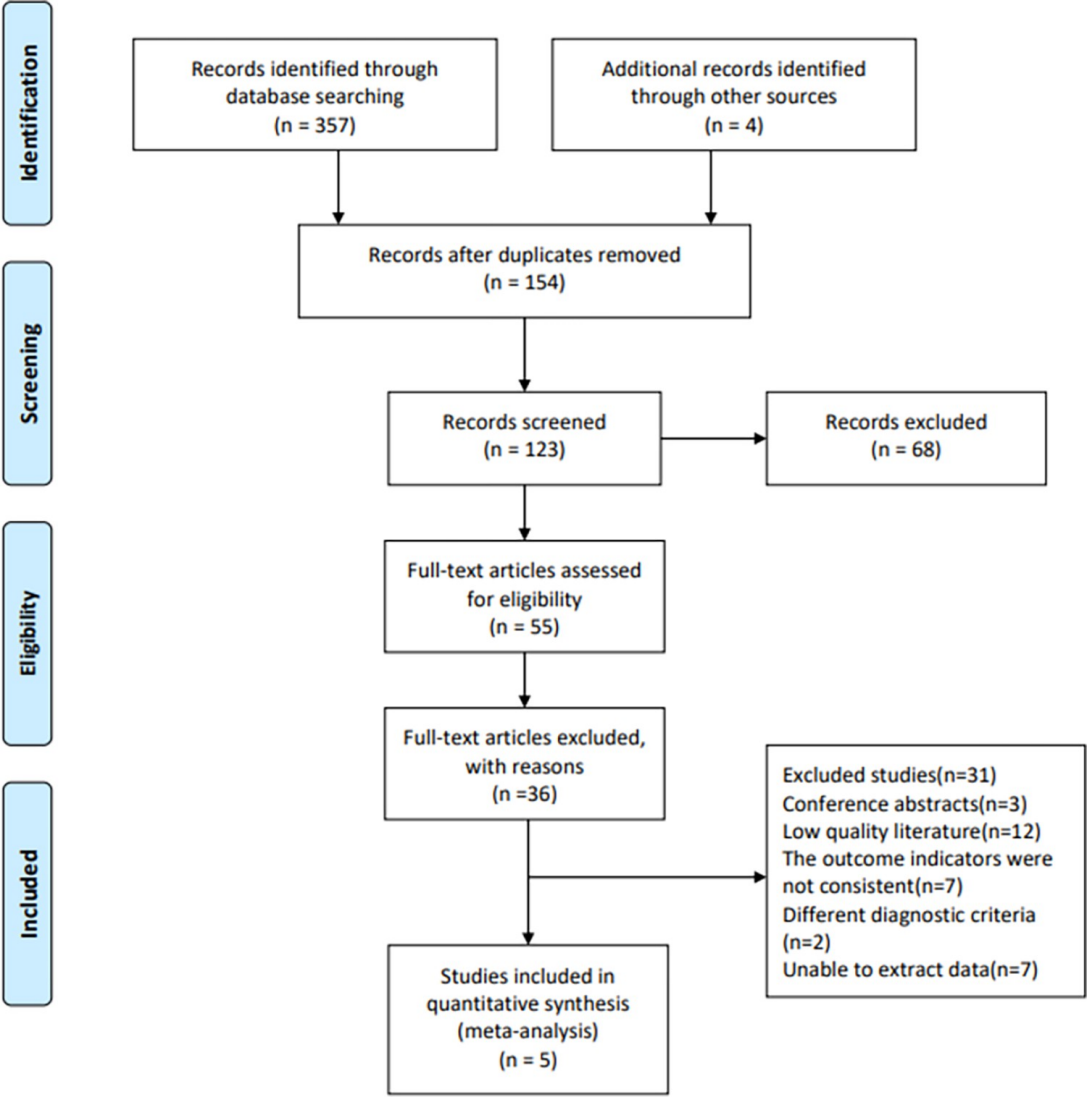
Flow diagram based on the Preferred Reporting Items for Systematic Reviews and Meta-Analyses (PRISMA) statement.

A total of 357 studies were obtained by searching electrical databases, and five trials [26–30] were finally included (Fig 1). There were 356 patients in total and involved for meta-analysis (152 aquatic physical therapy and 204 control group). A summary of characteristics of the included studies was shown in Table 1. All of the studies were published in English during 2012–2019. The studies were divided into 2 groups and were arranged as follow: Group1 aquatic physical therapy × usual care; Group 2 aquatic physical therapy × land-based exercise. The duration of the interventional programs was 8 weeks and 1 year.

**Table 1:** Characteristics of studies included in the meta-analysis.

First author, year	Country	Cancer stage	Experimental group			Control group			Outcomes measures
			sample size	age (year)	intervention	sample size	age (year)	intervention	
Cantarero-Villanueva I, 2012 [16]	Spain	I–IIIA	20	48.4 ±10.8	APT (aerobic and mobility exercises, 1 hour×3 per week for 8 weeks)	20	46.2 ±7.4	usual care	pressure pain threshold PFS, BMI waist circumference
Cantarero-Villanueva I, 2013 [27]	Spain	I–IIIA	32	49±7	APT (aerobic and endurance exercises, 1 hour×3 per week for 8 weeks)	29	47±8	usual care	PFS mood state abdominal and leg strength
Cuesta-Vargas AI, 2014 [28]	Spain	I–IIIA	22	47.27 ±6.57	MMPP+DWR (1 hour×3 per week for 8 weeks)	20	48.67 ±9.66	normal activities	PFS, SF-12 EuroQoL-5D EuroQoL-VAS
Fernández-Lao C, 2013 [29]	Spain	I–IIIA	33	48±7	APT (aerobic and strength exercises, 1 hour×3 per week for 8 weeks)	34	48±8	usual care	BMI, QoL
						31	49±8	land exercise (1hour×3 per week for 8 weeks)	waist circumference incidence of secondary lymphedema
Odynets T, 2019 [30]	Ukraine	I–II	45	58.84 ±1.36	APT (breathing, endurance and strength exercises, 1 hour×3 per week for 1 year)	40	59.40 ±1.24	pilates exercise	QoL was assessed using the FACT-B
						30	59.10 ±1.37	yoga exercise (1hour×3 per week for 1 year)	

APT: aquatic physical therapy, PFS: Piper Fatigue Scale, BMI: body mass index, MMPP: multimodal physiotherapy programme, DWR: deep water running, SF-12: Short Form 12, EuroQoL-5D: European Quality of Life five dimensions, EuroQoL-VAS: European Visual Analogue Scale, QoL: Quality of life, FACT-B: Functional Assessment of Cancer Therapy questionnaire with a specific module for breast cancer.

### Critical appraisal

**Fig 2:**
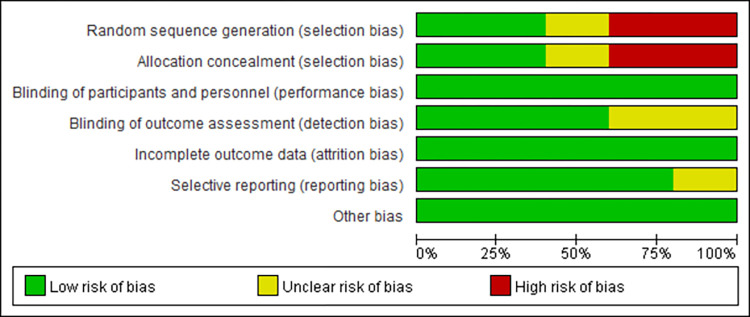
Risk of bias graph.

**Fig 3:**
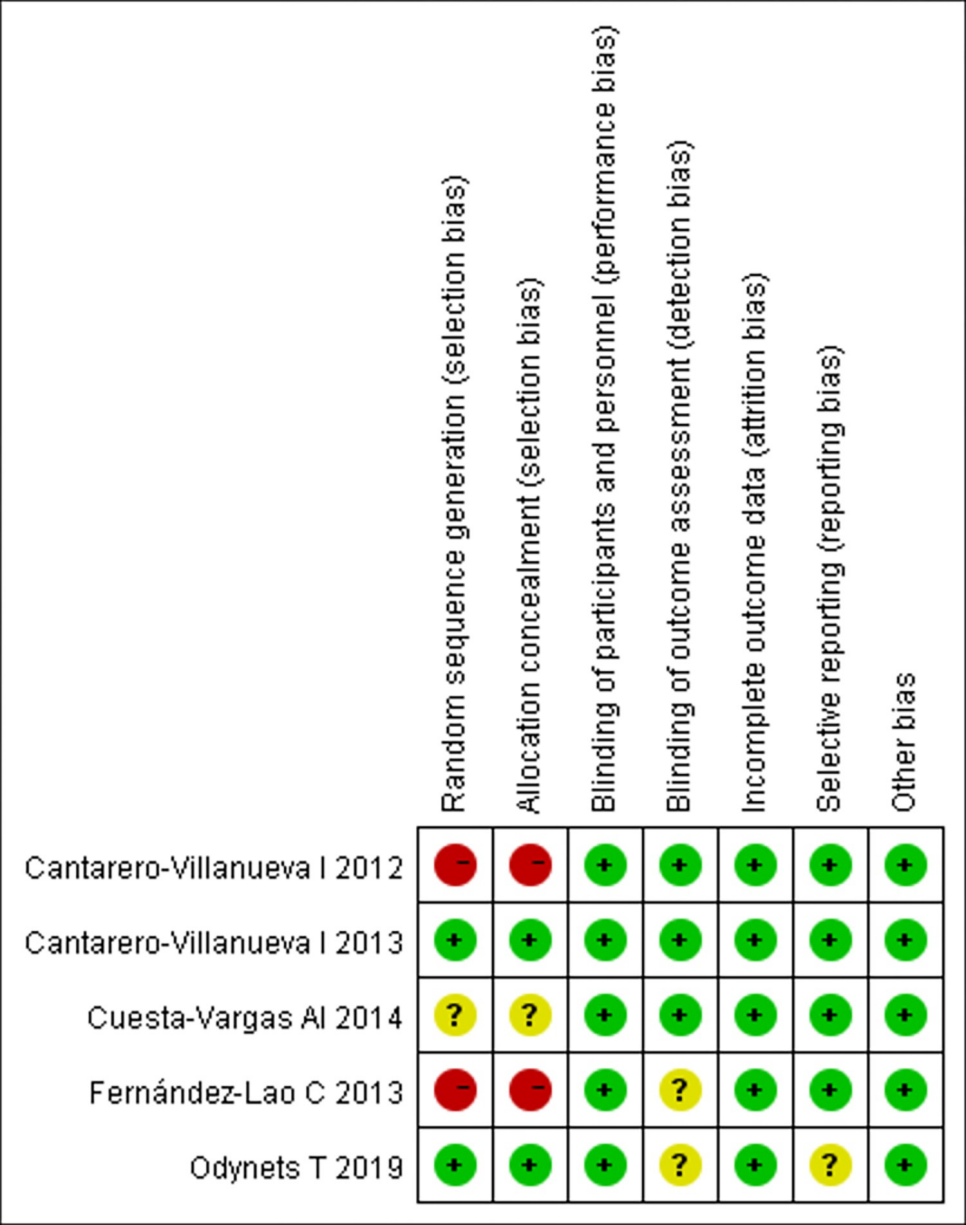
Risk of bias summary.

The results of quality assessment of the included studies by Cochrane Collaboration Handbook are shown in Figs 2 and 3. Where two had random sequence generation, two had allocation concealment, all trails had blinding of participants and personnel, three had blinding of outcome assessment, no trials were assessed to have incomplete outcome data, risk of selective reporting in four was low, and other bias in all trials was low.

### Effect of intervention

#### Aquatic physical therapy × usual care

**Fig 4:**
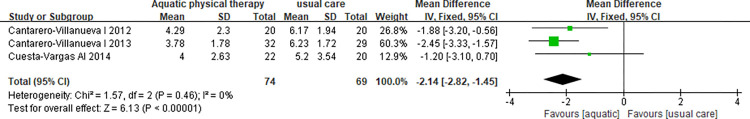
Meta-analysis of studies assessing fatigue between aquatic physical therapy and usual care.

*Fatigue*. A meta-analysis was made on three studies involving 143 patients with breast cancer [26–28]. The fixed effect model was used because heterogeneity was 0% (*p* = 0.46). There was a statistically significant difference (MD = -2.14, 95%CI: -2.82, -1.45, *p<*0.01) on fatigue between aquatic physical therapy and usual care (Fig 4).

**Fig 5:**
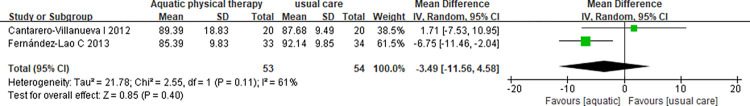
Meta-analysis of studies assessing waist circumference between aquatic physical therapy and usual care.

*Waist circumference*. A meta-analysis was made on two studies involving 107 patients with breast cancer [26, 29]. The random effect model was used because heterogeneity was 61% (*p* = 0.11). There was no statistically significant difference (MD = -3.49, 95%CI: -11.56,4.58, *p* = 0.4) on waist circumference between aquatic physical therapy and usual care (Fig 5).

#### Aquatic physical therapy × land-based exercise

**Fig 6:**
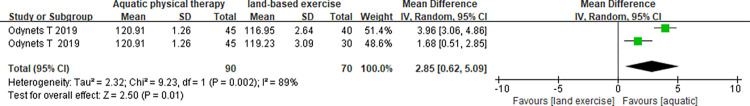
Meta-analysis of studies assessing QoL between aquatic physical therapy and land-based exercise.

*QoL*. Two studies were included involving 115 patients [30]. Because of heterogeneity, the random effects model was adopted (*p<*0.1, *I*^*2*^ = 89%*)*. The aquatic physical therapy has a statistically significant difference in improving the total score of Functional Assessment of Cancer Therapy questionnaire with a specific module for breast cancer (FACT-B) (MD = 2.85, 95%CI: 0.62, 5.09, *p* = 0.01), compared to the land-based exercise after 12 months intervention (Fig 6).

## Discussion

This systematic review and meta-analysis aimed to determine the effects of aquatic physical therapy on fatigue, QoL and physical index in patients with breast cancer. Based on the included RCTs (n = 5), we found that aquatic physical therapy significantly relieved fatigue and the effect size was moderate (*p<*0.01) compared with usual care. In addition, we also observed that, compared with land-based exercise, aquatic physical therapy greatly improved QoL in a large degree. However, we found that, compared with usual care, aquatic physical therapy cannot improve physical index (waist circumference). This may be due to the short intervention time, which is not enough to produce a significant statistical difference. Therefore, we concluded that aquatic physical therapy can reduce fatigue and improve QoL to a certain extent.

Fatigue is a common cancer-related symptom in breast cancer survivors [20] and caused by several factors, including cancer itself, chemotherapy, comorbidity, nutritional, and functional status [31]. This type of fatigue can’t be relieved by rest, it may actually worsen with decrease of physical activity [32], resulting in a harmful cycle. During aquatic exercising, oxygen consumption is 3 times greater at a given speed in water than on land, thus a training effect may be achieved at a significantly slower speed than on land [33]. This results in increased aerobic fitness for the patient more easily with a short time in water than on land. Meanwhile, the buoyancy force of the water reduces the stress on painful weight-bearing joints and facilitates shoulder movement [34]; the hydrostatic pressure of water increases in the range of motion (ROM) of shoulder flexion and abduction [35]. This helps improve the patient’s physical activity and relieve fatigue [33]. Our study demonstrated that aquatic physical therapy can have a significant effect on fatigue. Our results are in agreement with previous meta-analyses reporting on the positive effect of exercise on fatigue in this population [36, 37], as well as confirming the recommendations provided by the clinical guidelines about the effects of exercise on fatigue [7, 38].

In addition, women with breast cancer have an increased risk of developing depression, anxiety, sleep disturbances, and sexual dysfunction in the few months following their adjuvant chemotherapy and radiotherapy, which can lead to impairment of their QoL and a negative impact on domestic and social activities [30, 39]. It is found in this study that aquatic physical therapy consisting of various exercise modes―particularly endurance and strength exercises―is more effective for improving QoL associated with breast cancer treatment than land-based exercise, despite the similar intensity, frequency, and duration for the water- and land-based programmes during the exercise period. The reasons for this may be that performing exercises with women who had similar experiences created new friendships; interesting incidents that occurred during the exercise sessions, warm water, and pleasant music may have contributed to mood elevation [40]; a rational combination of breathing, endurance and strength exercises made it possible to use almost all the muscles of the body and improved emotional well-being [30]. Each one of the abovementioned reasons can potentially improve QoL. Therefore, aquatic physical therapy is crucial to address psychosocial issues and improve short and long term QoL outcomes in these patients.

Overall, it is suggested that women with breast cancer can derive health-related and clinical benefits by performing aquatic physical therapy within 5 to 6 months after surgery and who have completed adjuvant chemotherapy and radiotherapy. But aquatic physical therapy is not recommended for breast cancer patients with uncontrolled hypertension (diastolic pressure > 95 mmHg) [29] because the hydrostatic pressure during water exercise redistributes blood from the limbs to the thoracic cavity; this redistribution may have reduced the heart rate and transiently increased the blood pressure, which lead to unexpected risks like cardiovascular and cerebrovascular diseases for patients [41].

### Study limitations

A potential limitation of this study should be noted. The total number of patients participating in the meta-analysis is small that and we cannot solve the problems of standardization and heterogeneity in existing studies. Therefore, future research requires multi-center samples, larger sample sizes, and random methods to draw more robust conclusions from the current research. Additional research is needed to evaluate the benefits of aquatic physical therapy for other populations with cancer.

## Conclusions

In conclusion, this meta-analysis confirms that aquatic physical therapy reduces fatigue and improves QoL in women with breast cancer. The high level of adherence and lack of reported adverse effects in the included studies suggest that this is an acceptable programme that is safe and effective. Meanwhile, with the increasing prevalence of this disease and the growing number of breast cancer survivors, it is evident that greater efforts are needed toward improving health and physiological and psychological measures in this population. Our results give preliminary support to the implementation of aquatic exercise programs to promote the recovery of functional limitations following breast cancer treatment.

## Supporting information

S1 Checklist PRISMA 2009 checklist. (DOC)

S1 Appendix Search strategy. (PDF)
