# Supplementary material for: Does aquatic physical therapy affect the rehabilitation of breast cancer in women? A systematic review and meta-analysis of randomized controlled trials
Source: PLoS One. 2022 Aug 3;17(8):e0272337. doi: 10.1371/journal.pone.0272337 (PMC9348687; doi:10.1371/journal.pone.0272337)
Supplement: S1 Appendix — (PDF) [file pone.0272337.s002.pdf]

Appendix 1 Search strategy

PubMed

(("Hydrotherapy"[MeSH Terms] OR "aquatic exercise"[Title/Abstract] OR "aquatic therapy"[Title/Abstract] OR "aquatic*"[Title/Abstract] OR "aquatherapy"[Title/Abstract] OR "water based exercise"[Title/Abstract] OR "hydrokinesitherapy"[Title/Abstract] OR "water training"[Title/Abstract] OR "whirlpool bath"[Title/Abstract]) AND ("Breast Neoplasms"[MeSH Terms] OR "breast tumor"[Title/Abstract] OR "breast cancer"[Title/Abstract] OR "mammary cancer"[Title/Abstract] OR "malignant neoplasm of breast"[Title/Abstract] OR "breast malignant tumor"[Title/Abstract] OR "mammary carcinoma human"[Title/Abstract] OR "human mammary neoplasms"[Title/Abstract] OR "breast carcinoma"[Title/Abstract])) AND (randomized controlled trial [Publication Type] OR randomized[Title/Abstract] OR placebo[Title/Abstract])

Embase

('breast neoplasm':ab,ti OR 'breast tumor':ab,ti OR 'breast cancer':ab,ti OR 'mammary cancer':ab,ti OR 'breast malignant neoplasm':ab,ti OR 'breast carcinoma':ab,ti) AND ('hydrotherapy':ab,ti OR 'aquatic*':ab,ti OR 'water-based exercise':ab,ti OR 'hydrokinesitherapy':ab,ti OR 'water training':ab,ti) AND ('randomized controlled trial':ab,ti OR 'randomized':ab,ti OR 'placebo':ab,ti OR 'rct':ab,ti)
